# Mixed infection of an emaravirus, a crinivirus, and a begomovirus in *Pueraria lobata* (Willd) Ohwi

**DOI:** 10.3389/fmicb.2022.926724

**Published:** 2022-09-29

**Authors:** Xiaofei Liang, Shiqiang Mei, Haodong Yu, Song Zhang, Jiaxing Wu, Mengji Cao

**Affiliations:** National Citrus Engineering and Technology Research Center, Citrus Research Institute, Southwest University, Chongqing, China

**Keywords:** HTS, mechanical inoculation, *Pueraria lobata*, *Emaravirus*, mixed infection

## Abstract

*Pueraria lobata* (Willd) (*Pueraria montana var. lobata* (Willd.) Maesen & S. M. Almeida ex Sanjappa & Predeep) is an important herbal medicine used in many countries. In *P. lobata* plants showing symptoms of mosaic, yellow spots, and mottling, mixed infection of new viruses provisionally named *Pueraria lobata-*associated emaravirus (PloAEV, genus *Emaravirus*), *Pueraria lobata-*associated crinivirus (PloACV, genus *Crinivirus*), and isolate CQ of the previously reported kudzu mosaic virus (KuMV-CQ, genus *Begomovirus*) was confirmed through high-throughput sequencing. PloAEV has five RNA segments, encoding a putative RNA-dependent RNA polymerase, glycoprotein precursor, nucleocapsid protein, movement protein, and P5, respectively. PloACV has two RNA segments, encoding 11 putative proteins. Only PloAEV could be mechanically transmitted from mixed infected symptomatic kudzu to *Nicotiana benthamiana* plants. All three viruses were detected in 35 symptomatic samples collected from five different growing areas, whereas no viruses were detected in 21 non-symptomatic plants, suggesting a high association between these three viruses. Thus, this study provides new knowledge on the diversity and molecular characteristics of viruses in *P. lobata* plants affected by the viral disease.

## Introduction

High-throughput sequencing (HTS) technology enables the characterization of known plant viruses in a relatively short period and the discovery of previously unknown novel viruses in various plants (Cao et al., 2021; Liao et al., 2021; Zhang et al., 2021). It is a highly sensitive and unbiased method that can detect low-titered infections of both DNA and RNA viruses. Thus, HTS has emerged as a powerful method of detecting and identifying viruses, which are otherwise difficult to find using traditional methods (Wu et al., 2015).

The genus *Emaravirus* is the sole member of the family *Fimoviridae* (order *Bunyavirales*) with segmented, linear, single-stranded, negative-sense RNA genomes (Elbeaino et al., 2018). Viruses within the genus have 4–8 genomic RNA segments (some have up to 10 because of specific RNA variants) with reverse complementary 13 nucleotide (nt) stretches at 5′ and 3′ terminal ends that share high intra- and inter-species homology (Kubota et al., 2020; Kormelink et al., 2021). The type species is *Emaravirus sorbi* (common name, European mountain ash ringspot-associated virus, EMARaV) with six RNA segments encoding RNA-dependent RNA polymerase (RdRP, RNA1), glycoprotein precursor (GP, RNA2), nucleocapsid protein (NP, RNA3), putative P4 protein (RNA4), putative movement protein (MP, RNA5), and a P27 protein (RNA6), respectively (Benthack et al., 2005; Mielke and Muehlbach, 2007; von Bargen et al., 2019). As of April 2022, 24 species have been assigned to the genus, including *Emaravirus cajani* (pigeonpea sterility mosaic emaravirus 1, PPSMV-1) and *Emaravirus toordali* (PPSMV-2), which have been identified in *Leguminosae* plants (Elbeaino et al., 2014; Kumar et al., 2017).

The genus *Crinivirus* is included in the family *Closteroviridae*, order *Martellivirales* (Fuchs et al., 2020). The viruses in the genus contain two linear, positive-sense, single-stranded RNA totaling 15.6–17.9 kb. Most viruses have a bipartite genome, except the potato yellow vein virus (PYVV) with a tripartite genome (Livieratos et al., 2004; Fuchs et al., 2020). All molecules are needed for infectivity and are separately encapsidated. There are 14 species in the genus, most of which are whitefly-transmitted.

The genus *Begomovirus* contains DNA viruses with monopartite or bipartite circular, single-stranded genomes encapsidated in geminate particles (Brown et al., 2015). It is the largest genus of the family *Geminiviridae* with 445 species (Fiallo-Olive et al., 2021). Viruses in this genus are also transmitted by whiteflies.

China is one of the origins and distribution centers for *Pueraria* plants, where there are about 11 species (nine species and two varieties). Among them, *P. lobata* (Willd) (*Pueraria montana var. lobata* (Willd.) Maesen & S. M. Almeida ex Sanjappa & Predeep), commonly known as kudzu, is the most important and widely used *Pueraria* plant. Its dried roots, also called Ge-Gen, are used as Chinese herbal medicine to treat cardiovascular diseases, diabetes, and vascular hypertension (Wong et al., 2011; Song et al., 2014). With an emphasis on health and treatment of illness, kudzu is planted and used widely in China and other countries.

A wide range of pathogens, including viruses, have caused serious diseases in *Pueraria* plants, significantly affecting the production of Ge-Gen. However, the reported viruses [kudzu mosaic virus (KuMV, a begomovirus), tobacco ringspot virus (TRSV, a nepovirus), and soybean vein necrosis virus (SVNV, a tospovirus)] were all identified by traditional detection methods (Ha et al., 2008; Khankhum et al., 2013; Zhou et al., 2018). Therefore, further studies are needed to reveal whether there are other viruses affecting kudzu that might be responsible for commercial losses due to viral diseases.

With the aid of HTS, we identified mixed infections of three viruses (two new viruses and an isolate of KuMV) under the genera *Emaravirus, Crinivirus*, and *Begomovirus* in *P. lobata* plants. Then, the molecular and biological characteristics of these viruses were further studied.

## Materials and methods

### Plant materials

Leaf samples were collected from virus-like disease-affected *P. lobata* plants in limited areas of a hillside in Beibei, Chongqing, China, and total RNAs were used for HTS analysis. Other samples were collected in five different regions of Beibei, including Citrus village, Jiefangtai, Damotan, Jingangbei, and the campus of Southwest University.

### RNA preparation and HTS

The total RNA was extracted from 100 mg of composite leaf tissue of three different plants using Trizol reagent (Invitrogen, Thermo-Fisher, USA) as per the manufacturer's instructions. The RNA concentration was measured using a Nanodrop (Thermo Fisher Scientific, Waltham, United States), and the integrity was assessed using agarose gel electrophoresis. The ribosomal RNA (rRNA) was depleted with the RiboZero Magnetic Kit (Epicenter, Madison, United States), followed by rRNA-depleted cDNA library construction with TruSeq RNA Sample Prep Kit (Illumina, San Diego, CA, U.S.A.). The prepared libraries were then sequenced using an Illumina HiSeq X Ten platform (Mega Genomics Company, Beijing, China) to generate 150-bp paired-end reads.

### Data processing

Low-quality reads and adapters of output raw reads were removed using the ′Trim Reads…′ program with default parameters (quality limit 0.05 and the maximum number of ambiguities 2) within the software CLC Genomics Workbench 9.5 (Qiagen, Hilden, Germany). The remaining reads were *de novo* assembled into contigs by using the Trinity program with default parameters, which were then annotated based on a BLASTn and BLASTx search (https://blast.ncbi.nlm.nih.gov/Blast.cgi) against the viral (taxid:10239) sequence database of National Center for Biotechnology Information (NCBI). Finally, virus-associated contigs were used for further analyses.

### Virus genome determination

To obtain the complete sequences of the viral RNAs identified *in silico*, total RNAs were extracted from a single sample using a Trizol reagent. The 5′ and 3′ terminal sequences of the *Pueraria lobata-*associated emaravirus (PloAEV) and *Pueraria lobata-*associated crinivirus (PloACV) were obtained by rapid amplification of cDNA ends (RACE) using SMARTer RACE 5′/3′ Kit (Takara, CA, USA) with the gene-specific primers (Supplementary Table S1) designed using Primer Premier 5. The amplicons were sequenced to obtain the exact 5′ and 3′ ends of each segment. Then the full genome of PloAEV was amplified by one-step RT-PCR with primers designed based on the terminal sequence, using a PrimeScript One-Step RT-PCR Kit (Takara, Japan). The genome of PloACV was amplified in segments by PCR using PrimeSTAR Max DNA Polymerase (Takara, CA, USA) after reverse transcription with PrimeScript™ II 1st strand cDNA Synthesis Kit (Takara, CA, USA) using random 6 mers. As for the KuMV isolate CQ (KuMV-CQ), total DNAs were extracted using DNAzol Reagent (Invitrogen, Thermo-Fisher, USA), referring to the manufacturer's protocols. The genomes were then amplified with inverse PCR primer pairs. The elongation time was appropriately set according to the approximate length of sequences. All PCR amplicons were purified using Gel Extraction Kit (Omega Bio-Tek, Norcross, United States), cloned into the pEASY T1 vectors (TransGene, Beijing, China), and Sanger-sequenced by the Tsingke company (Beijing, China). The sequence of each amplicon was verified from at least five randomly selected clones and assembled using the SeqMan software (DNAStar, Madison, USA).

### Sequence and phylogenetic analyses

The full-length genomic sequences of the novel viruses were subjected to the ORF Finder (https://www.ncbi.nlm.nih.gov/orffinder/) to predict the open reading frames (ORFs) of each viral genomic RNA component and translate them into protein sequences. The potential functional and conserved domains of the encoded proteins were inferred from the NCBI conserved domain database (CDD) search tool (https://www.ncbi.nlm.nih.gov/Structure/cdd/docs/cdd_search.html) (Yang et al., 2020). Each protein was blasted against NCBI's non-redundant protein databases (nr) to find homologous viral protein sequences. The conserved motifs were found by the multiple sequence alignment of these viruses using the ClustalW. Transmembrane domains, N-glycosylation sites, and signal peptides were predicted using TMHMM-2.0 (https://services.healthtech.dtu.dk/service.php?TMHMM-2.0), NetNGlyc-1.0 (https://services.healthtech.dtu.dk/service.php?NetNGlyc-1.0), and SignalP-3.0 (https://services.healthtech.dtu.dk/service.php?SignalP-3.0), respectively. The pairwise identity of viral nucleotide and amino acid (aa) sequences were calculated with the ′Create Pairwise Comparison′ program of the CLC Genomics Workbench. The aa sequences of member species (RdRP, GP, and NP of emaravirus; RdRP, CP, and HSP70h of crinivirus) were obtained from the International Committee on Taxonomy of Viruses (ICTV) report and NCBI database.

Representative protein sequences were selected for constructing corresponding maximum-likelihood (ML) phylogenetic trees. *L-INS-i* option within MAFFT and *-htmlout* option (*-gt 0.8 -st 0.001 -cons 60*) within TrimAI (v. 1.2) were used to align the sequences and remove ambiguously aligned regions (Katoh and Standley, 2013). That is, all columns with gaps in more than 20% of the sequences or with a similarity score lower than 0.001 were removed, unless this removes more than 40% of the columns in the original alignment (Capella-Gutierrez et al., 2009). The ML phylogenetic trees were inferred using IQ-TREE (v 1.6.12) with 1,000 bootstrap replicates under the substitution models chosen according to the Bayesian Information Criterion (Minh et al., 2020). Fig-TREE (v 1.4.4) was used to visualize the phylogenetic trees.

### Field surveys

To investigate the percent infection of the three viruses, 35 symptomatic and 21 asymptomatic leaf samples were collected randomly from the five areas mentioned earlier from May to July 2021. After extracting the DNA and RNA of the samples, the PCR assays were developed for detecting the KuMV-CQ and the RT-PCR assays for the PloAEV and PloACV with the corresponding specific primers (Supplementary Table S1). PCR products were separated by electrophoresis on a 1.5% agarose gel which was stained using (BM) GelRed nucleic acid gel stain (Biomed, Beijing, China) and visualized under UV light.

### Mechanical inoculation

For mechanical inoculation, 0.5 g of leaf tissue was taken from a mixed sample of three infected *P. lobata* plants, homogenized in 1.5 mL of 50 mM sodium phosphate buffer (PBS, pH 7.4), and inoculated mechanically into a group of five 2-week-old plants of *Nicotiana benthamiana* by rubbing 150 μL of the sap onto the first true leaf, which was previously dusted with carborundum powder. Six groups of plants (a total of 30 plants) were each inoculated with the sap from three different plants of *P. lobata* (derived from a total of 18 source plants which were a subset of the 35 additional symptomatic plants from the field survey) and one group with PBS as negative controls. All the *N. benthamiana* plants were maintained in the greenhouse under controlled conditions. Total RNA and DNA were extracted from newly developed (systemic) leaves of *N. benthamiana* 20 days after inoculation (dpi). RT-PCR and RCR were conducted to test the infection of the three viruses, respectively.

## Results

### Virus identification

**Figure 1:**
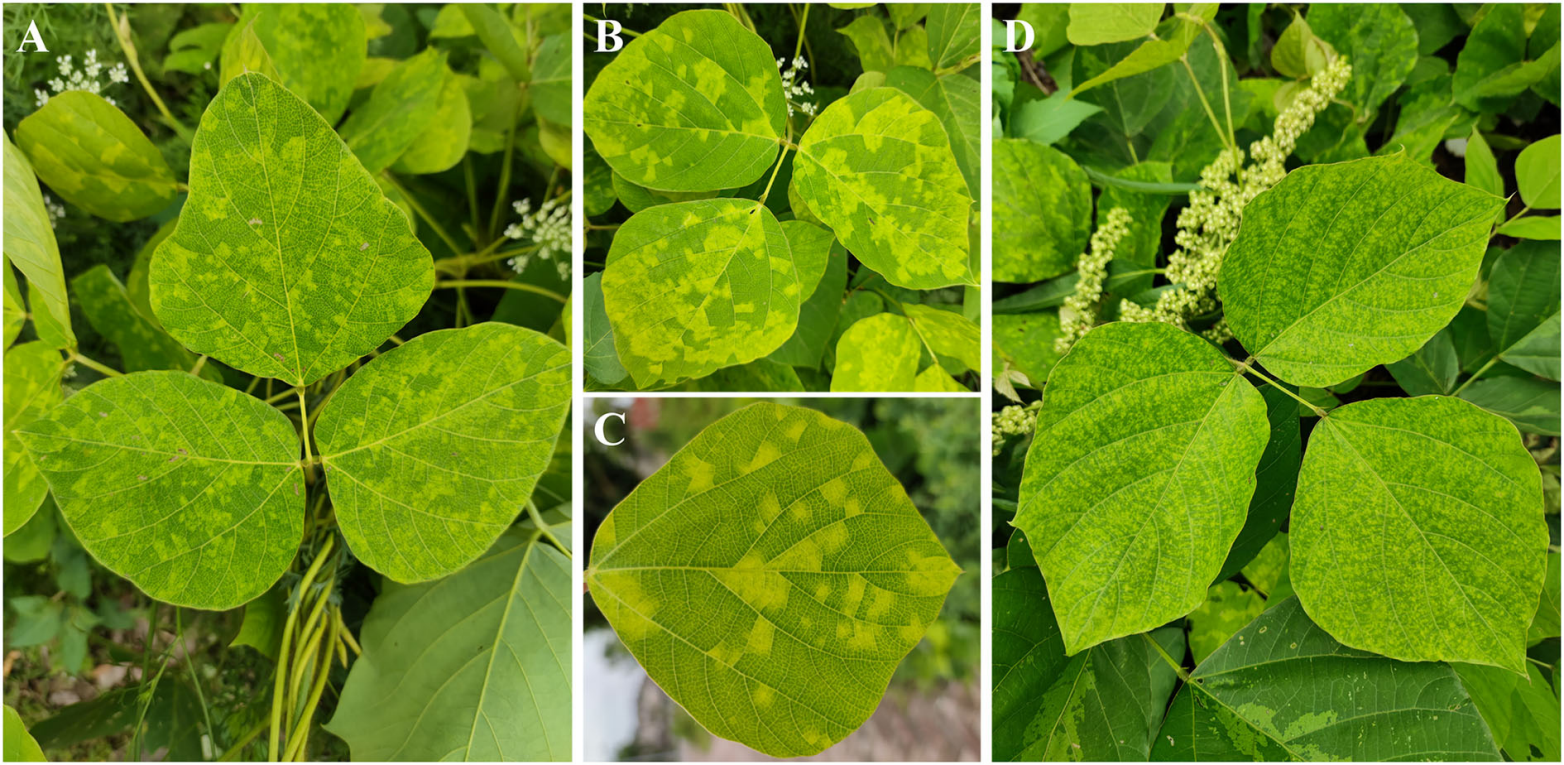
Symptoms observed on leaves of *Pueraria lobata* with a mixed infection of PloAEV, PloACV, and KuMV-CQ. The symptoms include yellow spots **(A)**, mosaic **(B,C)**, and mottling **(D)**.

To identify the putative viruses in diseased *P. lobata* leaves showing symptoms of yellow spots, mosaic, and mottling (Figure 1), the cDNA library was generated and sequenced, resulting in a dataset containing 65,477,710 raw reads. After filtering the low-quality sequences, the clean reads were assembled and yielded 106,721 contigs (including scaffolded regions). The contigs ranged in size from 200 to 50,780 bp. BLASTx analyses against the viral reference database identified 13 contigs (Table 1) exhibiting significant aa sequence identities to several different viruses, including nine contigs (1,238–7,100 nt) with high identities to five different genomic RNA segments of several members of the genus *Emaravirus*, two contigs (8,441 nt and 7,877 nt) with high identities to two distinct genomic RNA segments of members of the genus *Crinivirus*, and two contigs (1,188 nt and 2,481 nt) almost identical to DNA-A and DNA-B of a begomovirus, KuMV (Ha et al., 2008) (Table 1). The data suggested the potential presence of a novel emaravirus and a crinivirus, and variants of known KuMV in the sequenced sample. The viruses were tentatively named *Pueraria lobata-*associated emaravirus (PloAEV), *Pueraria lobata-*associated crinivirus (PloACV), and KuMV isolate CQ (KuMV-CQ). Then the 5′/3′ sequences of each segment were amplified by RACE using SMARTer RACE 5′/3′ Kit primers presented in Supplementary Table S1. Bands of corresponding size (near 300 bp) were selected and sequenced to confirm the terminal sequences.

**Table 1:** Analysis of HTS data from the diseased leaves of *P. lobate*.

**Virus**	**Number of contigs**	**Contigs**	**Consensus length**	**Percent identity^a^**	**Total read count^b^**	**Average coverage^c^**
PloAEV	9	RNA1	7,100		13,587	286.32
		RNA2	2,275		13,698	898.54
		RNA3a	1,596	86.39	8,147	728.92
		RNA3b	1,238		4,204	507.31
		RNA4a	1,481	90.75	2,753	277.76
		RNA4b	1,275		6,313	738.57
		RNA5a	1,882	67.69 (a and b)	16,232	1,292.40
		RNA5b	1,697	67.29 (a and c)	5,634	494.62
		RNA5c	1,709	83.82 (b and c)	950	83.14
PloACV	2	RNA1	8,441		3,211	56.98
		RNA2	7,877		6,578	125.01
KuMV-CQ	2	DNA-A	2,481		210	11.49
		DNA-B	1,188		67	3.71

a Percent nucleotide identity of contigs associated with the same RNA segment. The identities were calculated using the ′Create Pairwise Comparison...′ program of the CLC Genomics Workbench.

b Total number of reads in the targeted regions. Note that if a read is only partially inside a targeted region, it will still count as a full read.

c For each position in each target region, the coverage is calculated. The average coverage is calculated by taking the mean of all the calculated coverages in all the positions in all target regions. Note that bases in overlapping paired reads will only be counted as 1.

### Virus genome confirmations and analyses

**Figure 2:**
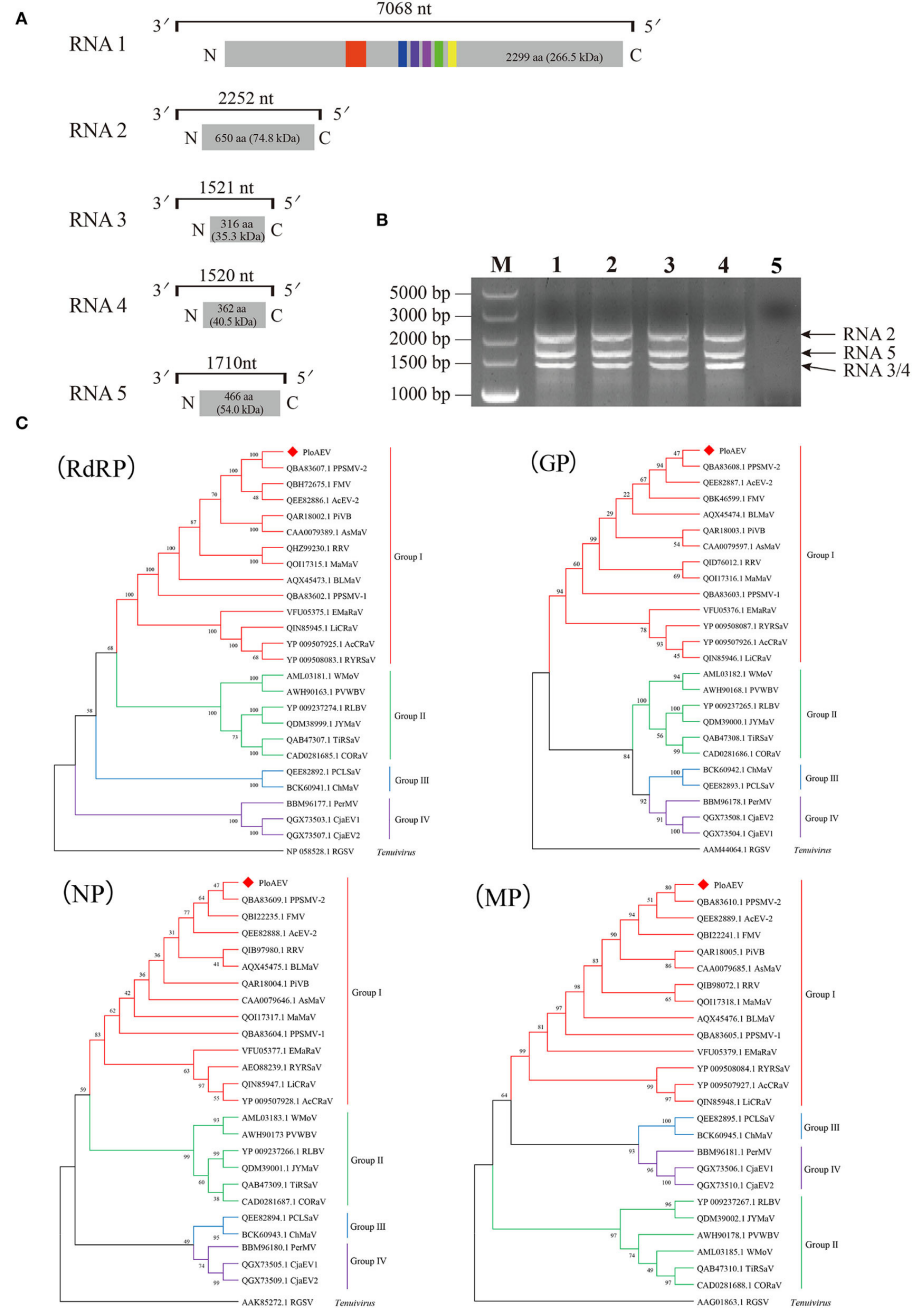
Genome and phylogenetic analysis of *Pueraria lobata*-associated emaravirus (PloAEV) identified in *P. lobata*. **(A)** Schematic genome organization of PloAEV. Genomic RNAs are displayed as a line, the boxes at whose ends indicate the 13 nts conserved at the RNA termini. Encoded open reading frames (ORFs) of each RNA segment are represented as gray rectangles with the amino acid (aa) length and predicted molecular mass (kDa) of proteins. Conserved motifs are shown in different colors. **(B)** Reverse transcription-polymerase chain reaction (RT-PCR) analysis of full-length RNA genomic segments of PloAEV. Sanger-sequencing showed that the bands corresponded to PloAEV RNA 2-5. Lanes 1-4, four different samples of infected leaves of *P. lobata*; Lane 5, mock of uninfected leaves; M, DL5,000 DNA Marker. **(C)** Phylogenetic trees were inferred using the aa sequences of the putative RdRP, GP, N, and MP. The trees were constructed using the maximum likelihood method with 1,000 bootstraps. The sequences of the corresponding protein of rice grassy stunt virus (genus *Tenuivirus*) were used as an outgroup. GenBank accession number of each sequence is shown. Bootstrap values are indicated at the nodes.

Interestingly, two or three contigs were associated with each of RNA3, RNA4, and RNA5 of PloAEV. The percent nucleotide identities of contigs associated with the same RNA segment were 86.39% (RNA3a and 3b), 90.75% (RNA4a and 4b), 67.69% (RNA5a and 5b), 67.29% (RNA5a and 5c), and 83.82% (RNA5b and 5c), respectively (Table 1), all of which could be detected in different samples. We suppose that there are three isolates of PloAEV. We detected one sequence of each (corresponding contigs 3a, 4b, and 5a) in a single sample with RNA1 and RNA2, while other contigs could not be detected (data not shown). Therefore, the RNA1, RNA2, and RNA3-5 associated with contigs 1, 2, 3a, 4b, and 5a, respectively, were presumed to be the same isolate of PloAEV. The genome of PloAEV, similarly to other emaraviruses, is presumed to be a negative-sense single-stranded RNA virus comprising five genomic segments (RNA1-5) (Figure 2A) that have been fully sequenced including the terminal 5′ and 3′ sequences. The full-length RNA1 of PloAEV (NCBI accession #ON181430) was amplified using one-step RT-PCR with the primers Emara-1-F/R (Supplementary Table S1). It is 7,068 nt long and codes a putative viral RdRP of 2,298 aa in length and approximately 266.5 kDa in predicted molecular mass by the complementary genome strand (nt position 127-7,023 of genome strand). It contains a domain of Bunya_RdRP superfamily (pfam04196, e-value: 1.00e-46) between aa positions 652 and 1,419. Sequence analyses revealed six conserved motifs [A (L_802_FYLGNKGLHGSPQELL), B (D_1129_ASKWSARD), C (S_1214_NWLQGN), D (H_1258_SDDS), E (K_1307_KTY), and F (K_1316_EFLS)] (Figure 2A), which are involved in the activities of RdRP catalysis, RNA binding, and cap-snatching (Kumar et al., 2017). The RNA2 (NCBI accession #ON181431) is 2,252 nt long with an ORF (nt 256-2,205) predicted to encode a putative GP (649 aa, 74.8 kDa) without any identifiable domain through CDD search but most related to emaraviruses. Like those of other emaraviruses, transmembrane helices (TMHs, aa 5-27, 111-133, 179-190, and 583-602) and N-glycosylation sites (N_65_VTC, N_204_STE, N_242_VSE, and N_331_KTE) were predicted for this putative GP (Supplementary Figures S1A,B). RNA3 (NCBI accession #ON181432) of 1,521 nt was identified to encode a putative NP (nt 473-1,420) required for encapsidation of viral RNAs with a predicted molecular weight of 35.3 kDa. Three conserved motifs (N_129_xSxNxxxA, N_178_xLA, and G_199_xE) were available, which may have a function in RNA binding (Wang et al., 2020). RNA4 (NCBI accession #ON181433) was 1,520 nt in size and contained an ORF (nt 353 to 1,438) encoding a putative movement protein of 361 aa with a predicted molecular mass of 40.5 kDa. The protein contains the conserved domain of the Emaravirus_P4 superfamily (pfam 16,505) between aa sites 13 and 361 predicted by CCD search. A signal peptide was also identified with a cleavage site (TSG_19_MK) using SignalIP-2.0. RNA5 (NCBI accession #ON181434) was determined from the RT-PCR product and sequenced, showing that it is 1,710 nt in length and contains an ORF (nt 222-1,619) encoding a putative P5 (465 aa and 54.0 kDa). The function of the encoded P5 is unknown because of the lack of any identifiable conserved domain. BLASTn and BLASTx searches against the NCBI database showed that PloAEV has the highest percent identities with PPSMV-2, and the nt (complete sequences) and aa identities from pairwise analyses were 52.29–72.62 and 44.26–81.65%, respectively (Table 2).

**Table 2:** The sequence percent identities on nucleotide (nt) and amino acid (aa) levels.

**Virus**	**The closest relative virus**	**Segments**	**Percent nt identity^a^**	**Percent aa identities^b^**
PloAEV	Pigeonpea sterility mosaic virus 2	RNA1	72.30	76.64 (RdRP)
		RNA2	69.22	66.77 (GP)
		RNA3	61.86	81.65 (NP)
		RNA4	72.62	80.39 (MP)
		RNA5	52.29	44.26 (P5)
PloACV	Lettuce chlorosis virus	RNA1	66.47	70.05 (RdRP)
		RNA2	64.40	86.33 (HSP70h) 74.40 (CP)
	Cucurbit chlorotic yellows virus	RNA1	67.35	70.83 (RdRP)
		RNA2	68.28	85.43 (HSP70h) 75.60 (CP)
KuMV-CQ	Kudzu mosaic virus	DNA-A	96.16	87.88–98.05
		DNA-B	83.84	92.19 (BC1) 92.95 (BV1)

a Percent nt identity of full RNA/DNA sequence with that of the closest relative virus.

b Percent aa identity of a protein encoded by ORF of the segment with that of the closest relative virus. Note that the data reported the proteins encoded by each segment (RdRP, GP, NP, MP, and P5) of PloAEV, the relevant gene products (RdRP, CP, and HSP70h) of PloACV, and a range of values for the DNA-A of KuMV-CQ. The identities were calculated using the ′Create Pairwise Comparison...′ program of the CLC Genomics Workbench.

The five RNAs of PloAEV are almost identical at the first 13 nucleotides of both terminal ends. The 5′ terminus (5′-AGUAGUGUU(AA)CUCC-3′) and 3′ terminus (5′-GGAGUUCACUACU-3′) of each segment are almost reverse complementary, which is a typical feature of emaraviruses and other negative single-strand RNA (-ssRNA) viruses. Therefore, primers designed in the conserved terminal sequences are expected to amplify all the PloAEV segments simultaneously. Specifically, we amplified three obvious bands using the primer Emara-5/3C from the total RNA of the mentioned sample (Figure 2B), and sequencing confirmed that these bands represented the segments of RNA2, RNA3/4, and RNA5, respectively. An RNA1-specific amplicon was not produced under the conditions used, possibly due to a low RNA1 titer or inadequate parameters to amplify this 7 kb RNA segment. Besides, a non-specific 500 bp band (not shown) may be caused by lower specificity of short primers.

**Figure 3:**
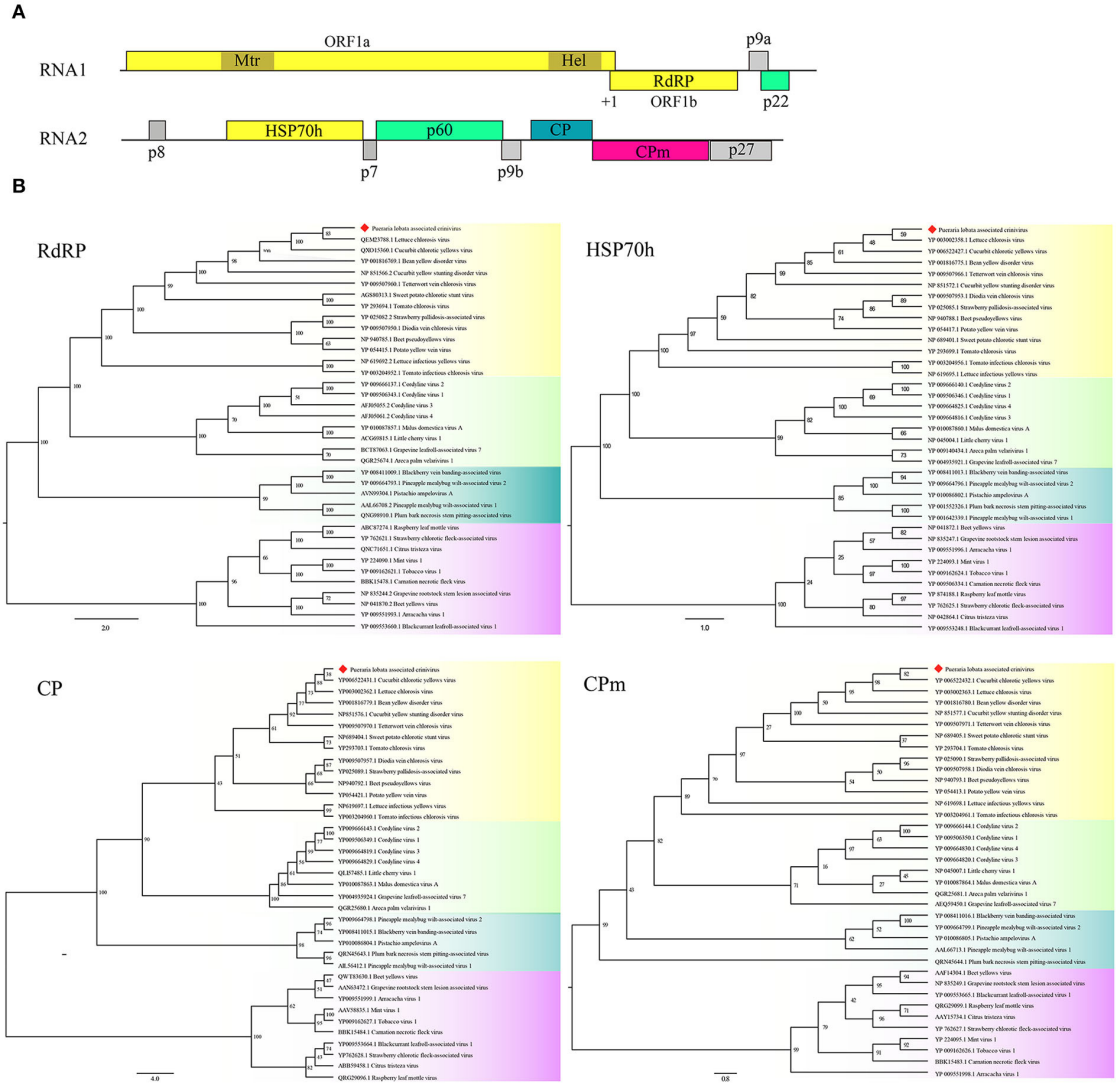
Genome and phylogenetic analysis of *Pueraria lobata*-associated crinivirus (PloACV) identified in *P. lobata*. **(A)** Schematic representation of the PloACV. The genomic RNA1 and RNA2 are presented by a line, with the ORFs indicated by colored boxes. ORFs are shown above or below the line, indicating that they are found in different reading frames. On RNA1, +1 indicates a putative +1 ribosomal frameshift site. Methyltransferase domain (Mtr) and RNA helicase domain (Hel) were predicted in ORF1. **(B)** Phylogenetic analysis of the amino acid sequences deduced for the RdRP, HSP70h, CP, and CPm of criniviruses (yellow boxes), velariviruses (pale green boxes), ampeloviruses (dark green boxes), and closteroviruses (red boxes) in family *Closteroviridae*. GenBank accession number of each sequence is shown. Bootstrap values are indicated at the nodes.

Two contigs from the assembly of 9,789 reads corresponded to RNA1 and RNA2 of a novel crinivirus, the PloACV (Table 1). The two genomic RNA segments were completely sequenced after 5′/3′ RACE and amplification using primers designed based on the contig sequences (Supplementary Table S1). The genome shared the highest percent identities with cucurbit chlorotic yellows virus (CCYV) at the nt level: 67.35% of RNA1 and 68.28% of RNA2 (Table 2). RNA1 (NCBI accession #ON181428) is 8,612 nt in length and contains three ORFs (Figure 3A). ORF1 is expressed by +1 ribosomal frameshift of ORF1a/1b like other criniviruses. ORF1a encodes a 1,994-aa protein (274.78 kDa) showing 70.05% aa identity with the RdRP of lettuce chlorosis virus (LCV) and 70.83% of CCYV (Table 2; Supplementary Figure S4). It contains two conserved domains associated with viral replication in *Closteroviridae*, a viral methyltransferase domain of Vmethyltransf superfamily (cl03298) between the aa sites 539-859 (*E*-value = 1.89e-24), and an RNA helicase domain of Viral_helicase 1 superfamily (cl26263) between the aa sites 1,697–1,960 (*E*-value = 1.69e-23). ORF1b (521 aa) encodes a putative RdRP of 60.72 kDa, which contains a conserved domain of RdRP_2 superfamily (pfam00978) between the aa sites 68-512 (*E*-value = 1.51e-125). ORF2 is 237 nt in length and encodes a predicted 9.27 kDa protein (P9a) of 78 aa that was predicted to have a signal anchor and a TMH but showed no significant sequence similarity to the corresponding proteins of other criniviruses. ORF3 is 588 nt long and encodes a predicted 159 aa protein (P22). The RNA2 (NCBI accession #ON181429) comprises 8,049 nt with 8 ORFs. Small ORF1, ORF2, ORF4, and ORF6 may encode 7.73-kDa protein (P8), 6.58-kDa protein (P7), 9.42-kDa protein (P9b), and 27.23-kDa protein (P27), respectively. BLAST and CD-search did not reveal any significant hits for these proteins in the databases. ORF3, ORF5, ORF7, and ORF8 may encode an HSP70h, P60 with a putative immunoglobulin-blocking virulence protein domain (cl37462, *E*-value = 1.04e-04), coat protein (CP), and coat protein minor (CPm), respectively, according to the corresponding conserved domains. The HSP70h and CP showed 86.33% and 74.40% identities with the corresponding proteins of LCV, and 85.43% and 75.60% with CCYV (Table 2).

A relatively small number of reads (277 reads) were assembled into two contigs corresponding to KuMV-CQ (Table 1). The genome was amplified using primers KuMV-A-Inv-F/R (DNA-A) and KuMV-B-Inv-F/R (DNA-B) and fully sequenced and found to have a bipartite genome with circular DNA-A (NCBI accession #ON181435) and DNA-B (NCBI accession #ON181436) that are 2,731 nt and 2,666 nt in length, respectively. The CQ isolate shared nucleotide sequence identity of 96.16% with a known KuMV isolate (DQ641690.1) in DNA-A and 83.84% with the isolate (DQ641691.1) in DNA-B (Ha et al., 2008) (Table 2). Typical genomic structures of begomoviruses could be found in KuMV-CQ DNA-A and DNA-B. DNA-A has six ORFs: two ORFs (AV1 and AV2) were encoded in sense, and four ORFs (AC1, AC2, AC3, and AC4) were encoded in antisense. DNA-B encoded two ORFs: BC1 in sense and BV1 in antisense. The intergenic region (common region, CR) encompassed a conserved stem-loop with the 5′-TAATATTAC-3′ sequence. The percent of aa identities of proteins encoded by DNA-A and DNA-B with KuMV ranged from 87.88 to 98.05% (Table 2).

### Phylogenetic analysis

Four phylogenetic trees were constructed to establish the relationships between PloAEV and other emaraviruses (Figure 2C) based on the amino acid sequences and using rice grassy stunt tenuivirus (RGSV) as an outgroup. Interestingly, PloAEV and PPSMV-2 were always grouped into one clade, despite bootstrap support of <50% for GP and N.

Phylogenetic analyses were performed on the amino acid sequences of the RdRPs, HSP70h, CPs, and CPms of criniviruses to confirm the relationships of PloACV with others in *Closteroviridae* (Figure 3B). The data indicated that PloACV consistently clustered with others in *Crinivirus* and was distinct from those in other genera. The closest relatives of PloACV confirmed by phylogenetic analyses were LCV and CCYV.

### Field surveys

To evaluate the percent infections of PloAEV, PloACV, and KuMV-CQ present in other plants, 56 additional *P. lobata* samples were collected from five areas in Beibei and detected by RT-PCR. Among the samples, 35 plants showed obvious mosaic and mottling symptoms, and 21 plants were asymptomatic. Interestingly, the results showed that the samples were either mixed-infected by the three viruses at the same time (35 symptomatic plants) or infected by none of them (21 asymptomatic plants, and part of the results are shown in Supplementary Figure S2). No plants in the fields with obvious symptoms were from single or double viral infections. Therefore, the symptoms caused by a single virus could not be characterized.

### Inoculation test

**Figure 4:**
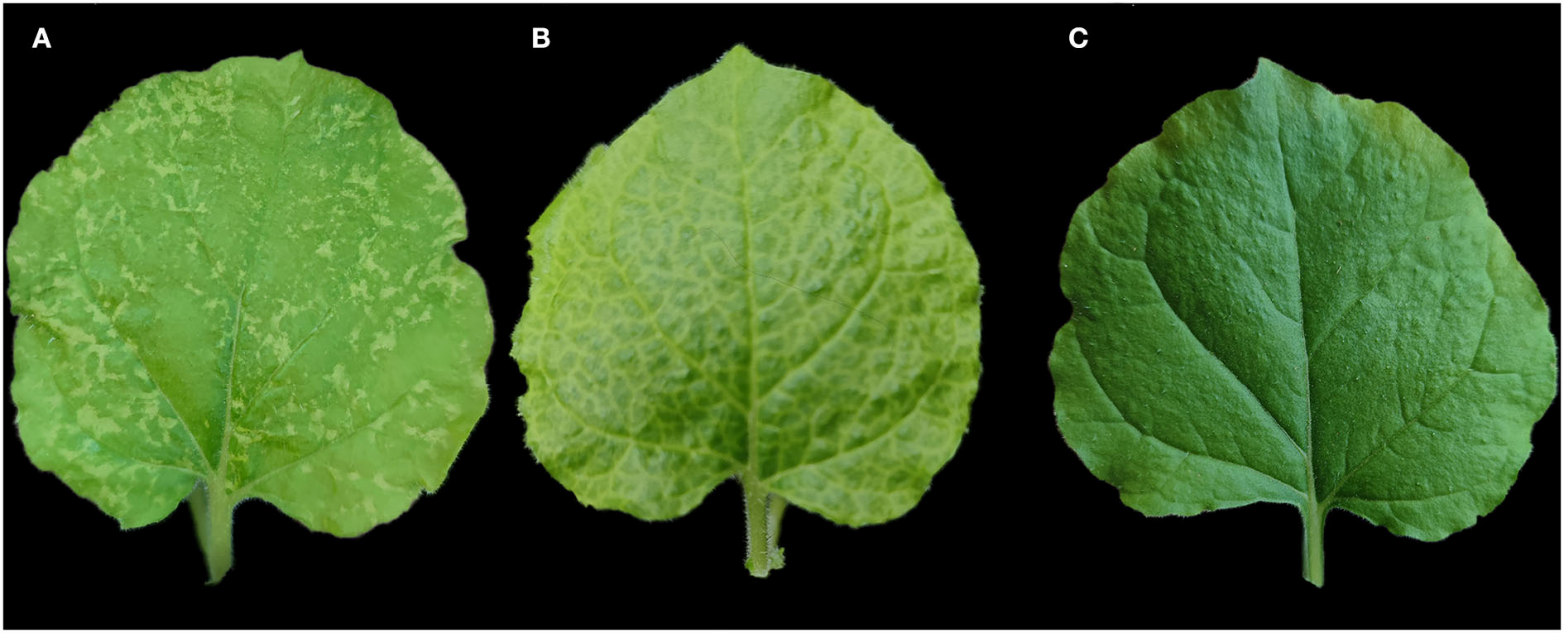
Mechanical inoculation of *Pueraria lobata*-associated emaravirus (PloAEV) to *Nicotiana benthamiana*. **(A)** The systemic leaf of *N. benthamiana* inoculated with sap from symptomatic *Pueraria lobata* leaves infected by the three viruses showed chlorotic spots. Treatment with sap from **(B)** leaves infected by potato virus X (PVX) and **(C)** inoculation buffer as the positive or negative control.

Thirty-two-week-old *N. benthamiana* plants were mechanically inoculated with sap from symptomatic *P. lobata* leaves infected by a mix of PloAEV, PloACV, and KuMV-CQ. At 16 dpi, chlorotic spots were observed on systemic leaves and expanded over the following days (Figure 4A). In contrast, as possible controls of the inoculation test, the leaves infected by potato virus X (PVX) showed vein clearing followed by obvious mosaic symptoms (Figure 4B). RT-PCR and PCR were conducted, respectively, to test systemic leaves from all inoculated plants using specific primers at 20 dpi (primer set Emara-det-F/R for PloAEV, Crini-RdRP-F1/R1 for PloACV, and KuMV-F1/R1 for KuMV-CQ). Only the PloAEV amplicons of the expected size were obtained from seven inoculated plants (Supplementary Figure S3). Subsequent sequencing of the amplification products confirmed the mechanical transmission of PloAEV to the *N. benthamiana* plants. PloACV and KuMV-CQ were not detected in the inoculated plants in this bioassay (data not shown).

## Discussion

*Pueraria* plants have a long history of being widely planted and used as medicine and food in Asia (Croom, 2004). However, over recent years, virus diseases have become a restrictive factor in *Pueraria* production. In 2008, a geminivirus, KuMV, was identified in *P. montana* (synonymous with *P. lobata*) affected by mosaic symptoms, which was the first virus identified in kudzu (Ha et al., 2008). After that, the isolate Yg3 of KuMV associated with the yellow vein mosaic symptom was identified by Zhang and Wu (2013) in China. In addition to the geminiviruses, it was also reported that TRSV and SYNV can infect kudzu (Khankhum et al., 2013; Zhou et al., 2018). In previous studies, all the viruses were detected by PCR using degenerate primers or ELISA tests and Sanger-sequencing but not by HTS. Therefore, it cannot be ruled out whether other plant viruses were associated with the symptoms.

Here, to understand the viral diversity of the *P. lobata* with symptoms of mottle and vein yellowing, we adopted HTS based on Illumina technology, coupled with metagenomic analysis. After a series of systematic data processes and molecular assays, we identified three viruses, including two new viruses and a virus with high homology with KuMV. Noticeably, some full genome segment sizes are shorter than the associated contigs, possibly caused by incorrect contig assembly due to improper parameters. From the molecular and phylogenetic perspectives, PloAEV and PloACV were consistent with those of all members in the genera *Emaravirus* and *Crinivirus*, respectively.

According to the species demarcation criteria of the ICTV, the amino acid sequence of relevant gene products of emaravirus and crinivirus should differ by more than 25% (Elbeaino et al., 2018; Fuchs et al., 2020). PloAEV shared >75% (and <81.65%) aa sequence identities with some recognized species in RdRP, NP, and MP genes, and <75% aa sequence identities with all known species in GP (≤ 66.77%) genes (Table 2). Therefore, PloAEV is likely to be a recombinant from three segments of PPSMV-2 and the other two segments of an unknown species. Thus, it is not unreasonable that PloAEV is a member of a new recombinant species of the genus *Emaravirus*. Besides, there are precedents of some extant *Emaravirus* species showing >75% aa sequence identities in some genes, such as PPSMV-2 and FMV (fig mosaic virus, *Emaravirus fici*) in the NP gene (80.06%, Supplementary Figure S4), and PPSMV-2 and AcV-2 (Actinidia virus 2, *Emaravirus kiwii*) in the MP gene (77.29%, Supplementary Figure S4). Moreover, PloAEV has fewer genomic RNA segments (five) than its closing relatives (PPSMV-2, FMV, and AcV-2). Our case provides new information that may be useful for modifying the current species demarcation criteria: in addition to aa identities, host plants of the virus, number of segments, transmission vectors, etc., should be also considered. Regarding the PloACV, the fact that HSP70h has high sequence homology (>75%) with other orthologs is not without precedent. The aa sequence of LCV gene product of HSP70h shared 85.97% identity with CCYV and 83.81% with bean yellow disorder virus (Supplementary Figure S4). Therefore, we suggest that PloAEV and PloACV should each be taxonomically classified as a member of a new species under the corresponding taxa, that is, viral genera *Emaravirus* and *Crinivirus*, respectively. Although the DNA-B of the CQ shares 83.84% nt sequence identity with that of an extant KuMV isolate, the comparison between DNA-A and B intergenic region showed that they belonged to the same virus. Similarly, high amino acid sequence identities of 92–97% which isolate CQ shared with other known KuMV isolates indicate that it is a new variant of KuMV.

*Pueraria lobata-*associated emaravirus has a multipartite genome of negative-sense monocistronic RNA components, with a stretch of almost reverse complementary sequences at the 5′ and 3′ termini of each, similar to those of emaraviruses (Kormelink et al., 2021). To ascertain if PloAEV had more than five segments, the full genome was amplified using the terminal primers Emara-5/3C. Except for the RNA2-5 identified by HTS, we did not find an additional RNA segment for PloAEV, supporting the penta-segmented nature of its genome. The absence of the gel band of RNA 1 when using primers Emara-5/3C may be due to the poor amplification stability of the polymerase. In contrast with RNA1-4, from which the putative proteins derived included recognizable functional domains, RNA5 encodes a protein of unknown function. It was not unusual in other emaraviruses that the role of P5 with a similar molecular weight but no sequence homology with each other is still to be determined (Buzkan et al., 2019; Wang et al., 2020; Fan et al., 2021; Rabbidge et al., 2021).

To investigate the mechanical transmissibility of PloAEV, PloACV, and KuMV-CQ, the sap of the infected leaves was mechanically inoculated to *N. benthamiana*. Contrary to some other emaraviruses that cannot be mechanically inoculated (Mielke-Ehret and Mühlbach, 2012; Nabeshima and Abe, 2021), the resulting inoculum successfully induced PloAEV infection and leaf chlorotic spots in *N. benthamiana*, which are often typical symptoms of emaraviruses (Zheng et al., 2017; Liu et al., 2020). Although most begomoviruses require either *Agrobacterium*-mediated transfer or biolistic delivery of cloned genomic DNA for their experimental transmission (Rojas et al., 2005), some are experimentally transmissible by mechanical inoculation (Chang et al., 2010). The lack of infection by KuMV-CQ may be because the titer is too low. It was not unexpected that PloACV was not mechanically transmitted, as members of the genus *Crinivirus* are phloem-restricted (Kiss et al., 2013).

Interestingly, PloAEV, PloACV, and KuMV-CQ were always found together in mixed infections in the *P. lobata* plants tested in our field survey. In plants showing obvious symptoms, no samples were infected with only one or two of the three viruses. As for the 21 asymptomatic plants, none of them tested positive for any of the three viruses. We have tried to use the infected *N. benthamiana* to back-inoculate kudzu plants, but the previous attempts have failed. Therefore, we could not characterize specific symptoms caused by each of the individual viruses so far. Although KuMV or KuMV-Yg3 were identified in the *P. montana* showing yellow vein mosaic disease (Ha et al., 2008; Zhang and Wu, 2013), there is still doubt that the symptom was caused by a single infection of the begomovirus.

Mixed infection modulates the development of symptoms in two ways, causing synergistic or antagonistic interactions (Syller, 2012). Some factors affect different reactions in plants, the suppression of one virus by another, or, conversely, the intensification of symptoms. The manifestation of a viral infection during mixed infection is also influenced by the host plant. Regarding the association of viruses with leaf symptoms, the milder symptoms on the *N. benthamiana* leaves only caused by the PloAEV may indicate that mixed infection with these viruses causes aggravation of the visual symptoms and greater severity of the disease on *P. lobata*. However, when the viral titer was considered on the same host, the interaction of these viruses may be antagonistic because the number of reads assigned to PloAEV was significantly higher than others. Considering the above findings, it still needs to be further verified on the same host in future experiments.

In conclusion, mixed infection with two novel viruses, PloAEV and PloACV, and a new isolate of KuMV, KuMV-CQ, was detected on *P. lobata*. We observed the symptoms of PloAEV through mechanical inoculation into *N. benthamiana*. The field survey showed that these viruses had spread widely, which needs to be considered, and the potential mechanical and vector transmissibility should also be cause for concern.

## Data availability statement

The datasets presented in this study can be found in online repositories. The names of the repository/repositories and accession number(s) can be found below: https://www.ncbi.nlm.nih.gov/genbank/, ON181428; https://www.ncbi.nlm.nih.gov/genbank/, ON181429; https://www.ncbi.nlm.nih.gov/genbank/, ON181430; https://www.ncbi.nlm.nih.gov/genbank/, ON181431; https://www.ncbi.nlm.nih.gov/genbank/, ON181432; https://www.ncbi.nlm.nih.gov/genbank/, ON181433; https://www.ncbi.nlm.nih.gov/genbank/, ON181434; https://www.ncbi.nlm.nih.gov/genbank/, ON181435; https://www.ncbi.nlm.nih.gov/genbank/, ON181436; https://www.ncbi.nlm.nih.gov/genbank/.

## Author contributions

MC conceived and designed the experiments. XL, SM, and HY collected the samples and conducted the experiments. XL, SZ, and JW analyzed the data. MC, XL, and SZ discussed the results and drafted and revised the manuscript. All authors read and approved the final draft of the manuscript.

## Funding

This study was supported by the National Natural Science Foundation of China (No. 32072389), Innovation Research 2035 Pilot Plan of Southwest University (SWU-XDPY22002), Overseas Expertise Introduction Center for Discipline Innovation (111 Project) (B18044), and Chongqing Postgraduate Research Innovation Project (CYS21141).

## Conflict of interest

The authors declare that the research was conducted in the absence of any commercial or financial relationships that could be construed as a potential conflict of interest.

## Publisher's note

All claims expressed in this article are solely those of the authors and do not necessarily represent those of their affiliated organizations, or those of the publisher, the editors and the reviewers. Any product that may be evaluated in this article, or claim that may be made by its manufacturer, is not guaranteed or endorsed by the publisher.

## Supplementary material

Supplementary Figure S1 Transmembrane helices [TMHs, **(A)**] and N-glycosylation sites **(B)** predicted in glycoprotein precursor (GP) of *Pueraria lobata*-associated emaravirus (PloAEV). Four TMHs (aa 5-27, 111-133, 179-190, and 583-602) and four N-glycosylation sites (N65VTC, N204STE, N242VSE, and N331KTE) were predicted.

Supplementary Figure S2 Detection of *Pueraria lobata*-associated emaravirus (PloAEV), *Pueraria lobata*-associated crinivirus (PloACV), and kudzu mosaic virus-CQ (KuMV-CQ) in kudzu plants using (RT)-PCR assay. M, DL 2,000 DNA Marker; N, negative control. Lanes 1-25, 25 samples collected in different areas. The actual sizes of the PCR products are shown on the right side.

Supplementary Figure S3 RT-PCR detection of *Pueraria lobata*-associated emaravirus (PloAEV) in inoculated *Nicotiana benthamiana* using primer set Emara-det-F/ R. M, DL 2,000 DNA Marker; P, positive control; N, negative control; Lanes 1-10, part of inoculated *N. benthamiana* samples. The actual sizes of the PCR products are shown on the right side.

Supplementary Figure S4 Pairwise comparisons of relevant amino acid sequences generated from the member species of *Emaravirus* **(A)** and *Crinivirus* **(B)**. A, Comparisons of RNA-dependent RNA polymerase (RdRP), glycoprotein precursor (GP), and nucleocapsid protein (NP) of 24-member species of *Emaravirus* (plus PloAEV). B, Comparisons of RdRP, coat protein (CP), and HSP70h of 14-member species of *Crinivirus* (plus CCYV and PloACV). Only CP sequences of abutilon yellows virus are available. The values above the diagonal show the percent amino acid identities, while the values below the diagonal show the number of alignment positions where one sequence is different from the other.
