# Supplementary figures and images for: Mixed infection of an emaravirus, a crinivirus, and a begomovirus in Pueraria lobata (Willd) Ohwi
Source: Front Microbiol. 2022 Sep 29;13:926724. doi: 10.3389/fmicb.2022.926724 (PMC9557060; doi:10.3389/fmicb.2022.926724)

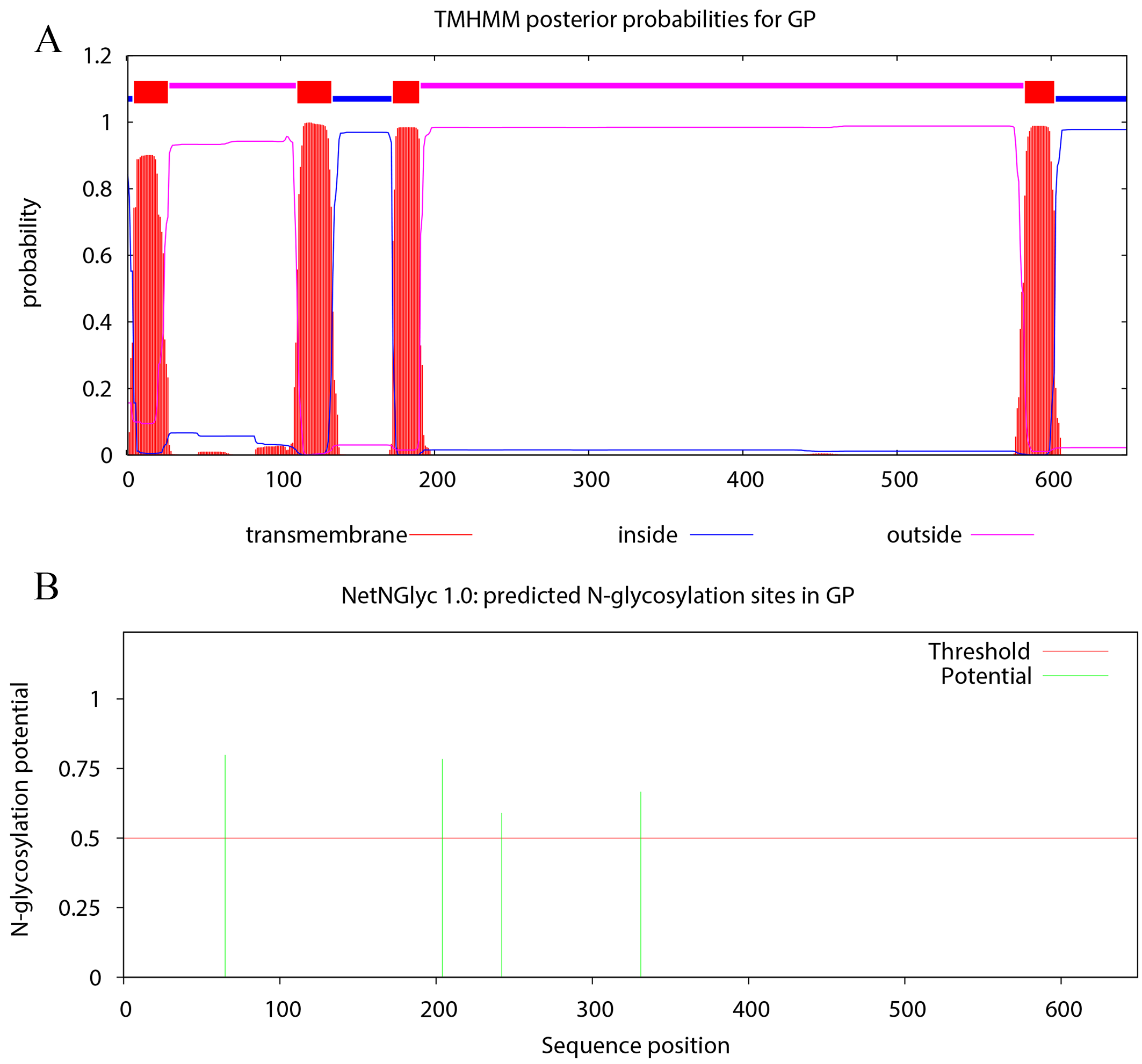

Supplement: Supplementary Figure S1 — Transmembrane helices [TMHs, (A)] and N-glycosylation sites (B) predicted in glycoprotein precursor (GP) of Pueraria lobata-associated emaravirus (PloAEV). Four TMHs (aa 5-27, 111-133, 179-190, and 583-602) and four N-glycosylation sites (N65VTC, N204STE, N242VSE, and N331KTE) were predicted. [file Image_1.TIF]

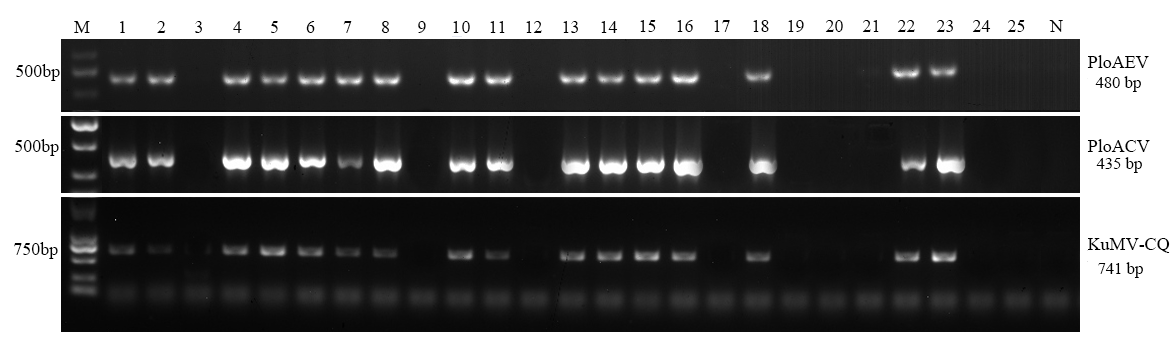

Supplement: Supplementary Figure S2 — Detection of Pueraria lobata-associated emaravirus (PloAEV), Pueraria lobata-associated crinivirus (PloACV), and kudzu mosaic virus-CQ (KuMV-CQ) in kudzu plants using (RT)-PCR assay. M, DL 2,000 DNA Marker; N, negative control. Lanes 1-25, 25 samples collected in different areas. The actual sizes of the PCR products are shown on the right side. [file Image_2.TIF]

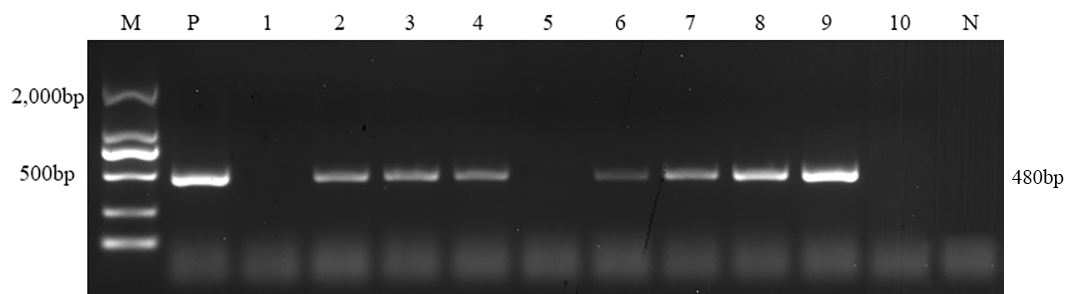

Supplement: Supplementary Figure S3 — RT-PCR detection of Pueraria lobata-associated emaravirus (PloAEV) in inoculated Nicotiana benthamiana using primer set Emara-det-F/ R. M, DL 2,000 DNA Marker; P, positive control; N, negative control; Lanes 1-10, part of inoculated N. benthamiana samples. The actual sizes of the PCR products are shown on the right side. [file Image_3.TIF]

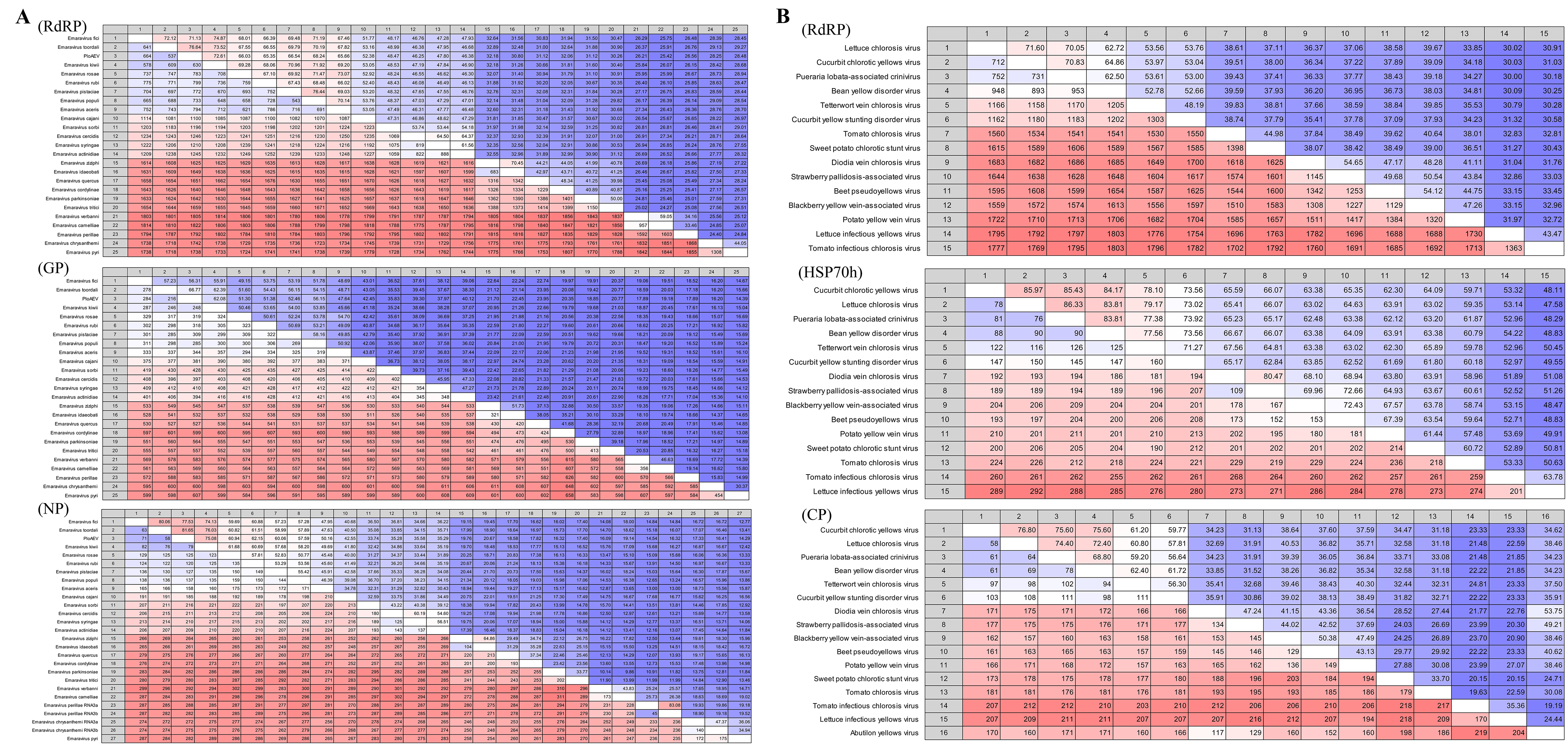

Supplement: Supplementary Figure S4 — Pairwise comparisons of relevant amino acid sequences generated from the member species of Emaravirus (A) and Crinivirus (B). A, Comparisons of RNA-dependent RNA polymerase (RdRP), glycoprotein precursor (GP), and nucleocapsid protein (NP) of 24-member species of Emaravirus (plus PloAEV). B, Comparisons of RdRP, coat protein (CP), and HSP70h of 14-member species of Crinivirus (plus CCYV and PloACV). Only CP sequences of abutilon yellows virus are available. The values above the diagonal show the percent amino acid identities, while the values below the diagonal show the number of alignment positions where one sequence is different from the other. [file Image_4.TIF]
