# Supplementary material for: Mixed infection of an emaravirus, a crinivirus, and a begomovirus in Pueraria lobata (Willd) Ohwi
Source: Front Microbiol. 2022 Sep 29;13:926724. doi: 10.3389/fmicb.2022.926724 (PMC9557060; doi:10.3389/fmicb.2022.926724)
Supplement: Supplementary file 5 [file Table_1.docx]

**Table S1** Primers used in the research

| Primers | Sequence | Note |
| --- | --- | --- |
| Emara-1-5′RACE | AGTGTAACCTGCTTCTTGGAG | RACE primers for PloAEV |
| Emara-1-3′RACE | TGTGATGGGATGCTGTTG |  |
| Emara-2-5′RACE | CACAAGCTGGGTAATTTCATC |  |
| Emara-2-3′RACE | ACTAAGCCACAGTAAAGACAC |  |
| Emara-3-5′RACE | TAGCACCAGAGCCAAGTAGAA |  |
| Emara-3-3′RACE | TCCTTTGAAATCGGCAATGGTGT |  |
| Emara-4-5′RACE | TATCATCTGGCAATGTCTTCC |  |
| Emara-4-3′RACE | ATACAAGAATGGCAAACACTG |  |
| Emara-5-5′RACE | TGTATTAGACCTCCGAACTGC |  |
| Emara-5-3′RACE | GGTATAGTTATTGATCGCATAG |  |
| Emara-5/3C | AGTAGTGWWCTCC^1^ | Conserved primers |
| Emara-1-F | AGTAGTGTTCTCCCTTTAATAATTATAATC |  |
| Emara-1-R | AGTAGTGAACTCCCTTTAATACAATACAAG |  |
| Emara-det-F | TGCCGATTTCAAAGGAGCCA | Detection primers target RNA3, 480 bp |
| Emara-det-R | GGGTCTCACTGCTGGGAATC |  |
| Crini-1-5′RACE | GAAATCAAACTTTCCTTCGTACG | RACE primers for PloACV |
| Crini-1-3′RACE | CCGATGATTTGATGGAGGATTTAAG |  |
| Crini-2-5′RACE | TGCCGTTATTGCCAAACAGGTCGGGATA |  |
| Crini-2-3′RACE | AGCCGTACTTAAACTGTCAGATG |  |
| RNA1-F1-121 | ATGTGGTCTGGCTTACTTCTTG | Verification of the PloACV sequences |
| RNA1-R1-1316 | CTAATGCGAAGGCTGGTATCC |  |
| RNA1-F2-1027 | CTTAACTACGCCTAAGCCTCAG |  |
| RNA1-R2-2319 | AACACTACGCCATCAACTTCAA |  |
| RNA1-F3-2298 | TTGAAGTTGATGGCGTAGTGTT |  |
| RNA1-R3-3650 | CTTGTGATGTCTTGCGTCTTATAC |  |
| RNA1-F4-3388 | ACGAGCAGCAAGCACCAT |  |
| RNA1-R4-5608 | GCCAGCAGCATCAGTTAATGT |  |
| RNA1-F5-5393 | GGTGAATGGTTAGCAGTGATGA |  |
| RNA1-R5-7339 | TCTCTTCTCCTGATTGGTGATGA |  |
| RNA1-F6-7315 | TGTCATCACCAATCAGGAGAAGA |  |
| RNA1-R6-8211 | TGAACTAAGCAATGGAGGAACTATG |  |
| RNA2-F1-117 | GCAAGCGTCACTCAATCTCA |  |
| RNA2-R1-1766 | ATATAGCAGCAGCGGATGGT |  |
| RNA2-F2-1507 | ATTGGATTGGAACGGTGTGAAT |  |
| RNA2-R2-2737 | AGTAGCAGTCTCATCAACATTGG |  |
| RNA2-F3-2296 | TTCAATGTCGCACGCTCAG |  |
| RNA2-R3-4068 | AGGAGGAAGGATGTCACTTGT |  |
| RNA2-F4-3539 | TGTGTAACTCTCAAGGCAGATT |  |
| RNA2-R4-5251 | TCATCGGCACTAGGCTCAC |  |
| RNA2-F5-5144 | AGCAGATGTAATGACCGAAGAAC |  |
| RNA2-R5-6999 | TCCGTGACTAAATAAGCGAACTC |  |
| RNA2-F6-6571 | TGAAGTGAACACGGCTATCG |  |
| RNA2-R6-7503 | GCTCAGTAACAAACCATAGAACC |  |
| Crini-RdRP-F1 | CTTCGTGCTTCAGTAGACCATCA | Detection primers, 435 bp |
| Crini-RdRP-R1 | AGTCCAACCACTCACCAATTCT |  |
| KuMV-A-Inv-F | TTTGTTGAACGCCTTTCC | Full sequence of KuMV-CQ DNA-A |
| KuMV-A-Inv-R | CACATGTTTAAAGTAAAGC |  |
| KuMV-B-Inv-F | ACTACGCTACGCAGCAGCC | Full sequence of KuMV-CQ DNA-B |
| KuMV-B-Inv-R | TACCCTCCCAGGGGTACAC |  |
| KuMV-F1 | ACCGGATGGCCGCGCTTCGGTGTCC | Detection primers, DNA-A, 741 bp |
| KuMV-R1 | CCATTGGCGTACCATAAGGTCGCCT |  |

^1^ W=A/T
